# Ecological Risk and Human Health Assessment of Heavy Metals in Sediments of Datong Lake

**DOI:** 10.3390/toxics13070560

**Published:** 2025-06-30

**Authors:** Gao Li, Rui Chen, Zhen Li, Xin Wu, Kui Xiang, Chiheng Wang, Yi Peng

**Affiliations:** Changsha General Survey of Natural Resources Center, China Geological Survey, Ningxiang 410600, China; ligao20226@163.com (G.L.); chenrui23@mail.cgs.gov.cn (R.C.); z846671288@163.com (Z.L.); wuxin@mail.cgs.gov.cn (X.W.); xiangkui@mail.cgs.gov.cn (K.X.); wangchiheng2024@163.com (C.W.)

**Keywords:** sediment, heavy metals, Monte Carlo modeling, ecological risk assessment, human health risk assessment

## Abstract

Heavy metal pollution of lake sediments is one of the prominent ecological and environmental problems worldwide, and it is of great significance to conduct research on heavy metal pollution in lake sediments to protect the ecological environment, safeguard human health, and promote sustainable development. As an integral part of Dongting Lake, Datong Lake holds a crucial ecological position. More than 10 years ago, due to a series of factors, including excessive fertilizer application and fishing, the water quality of Datong Lake declined, resulting in varying degrees of contamination by Cd, Mn, and other heavy metals in the sediments. After 2017, Datong Lake began to establish a mechanism for protecting and managing the lake, and its ecological and environmental problems have been significantly improved. To clarify the current situation of heavy metal contamination in the sediments of Datong Lake, 15 sediment samples were collected from the lake, and the contents of soil heavy metals Cd, As, Pb, Cr, Cu, Mn, Ni, and Zn were determined. A Monte Carlo simulation was introduced to carry out the ecological and human health risk evaluation of the sediments in the study area to overcome the problem of low reliability of the results of ecological and human health risk evaluation due to the randomness and incompleteness of the environmental data as well as the differences in the human body parameters. The results and conclusions show that (1) the average values of Cd, Pb, Cr, Cu, Mn, Ni, and Zn contents in the sediments of Datong Lake are higher than the background values of soil elements in the sediments of Dongting Lake, and the average values of As contents of heavy metals are lower than the background values of the soil, and the heavy metal contamination in the sediments in the study area is dominated by slight contamination, and the possibility of point-source contamination is slight. (2) The results of both the Geo-accumulation index and Enrichment factor evaluation showed that the degree of heavy metal contamination of sediments was Ni > Cu > Cr > Mn > Cd > Pb > Zn > As. (3) The average value of the single ecological risk index of heavy metal elements, in descending order, was as follows: Cd > As > Pb > Cu > Ni > Cr > Zn > Mn; all the heavy metal elements were at the level of light pollution, and the average value of the comprehensive ecological risk index was 32.83, which is a slight ecological risk level. (4) Both non-carcinogenic and carcinogenic risks for all populations in the study area remain low following heavy metal exposure via ingestion and dermal pathways. Ecological and health risk assessments identified As and Cd as exhibiting significantly higher sensitivity than other heavy metals. Consequently, continuous monitoring and source-tracking of these elements are recommended to safeguard long-term ecological integrity and public health in the region.

## 1. Introduction

Heavy metals are metals with a density greater than 4.5 g/cm^3^, which accumulate in the human body to a certain extent and cause chronic poisoning. Heavy metals are characterized by their high resistance to degradation and tendency to bioaccumulate [1,2]. They are widely present in aqueous environmental media and pose serious hazards to both the ecological environment and human health [3,4]. With the development of industry and agriculture, as well as the intensification of human activities, heavy metals such as Cd and Zn are continuously entering lakes, rivers, and wetlands through various channels, leading to the deterioration of the ecological environment [5,6,7]. As seen from recent studies related to heavy metal pollution, heavy metal pollution of lake sediments is one of the prominent environmental problems worldwide [8,9,10,11,12,13]. By analyzing the pollution status of 10 heavy metals in the sediments of 289 rivers and 133 lakes in 6 continents from 1970 to 2018, Niu et al. [14] showed that there was a significant increasing trend in the heavy metal contents of Pb, Hg, Cr, and Mn in the lake sediments and that the average metal concentrations in Europe and North America were generally higher than those in Africa, Asia, and South America. Research has shown [15,16] that sediments are the primary “source” and “confluence“ of heavy metals and other pollutants in lake water environments. This is because sediments contain a large amount of natural organic matter, microorganisms, iron and manganese oxides, and minerals [17,18,19]. These substances can bind with heavy metals through physical and chemical processes such as adsorption, complexation, and precipitation [20,21,22,23,24]. Currently, domestic and foreign experts and scholars have proposed various methods for studying sediment heavy metal pollution [25,26,27,28,29]. Subsequently, a variety of heavy metal ecological risk evaluation models have been developed based on pollution evaluation [30,31,32,33,34,35,36,37,38,39], and scholars have utilized these methods to research some typical lakes and have grasped the contamination characteristics, spatial distribution, and ecological risk level of heavy metals in lake sediments [40,41,42,43,44]. Further, a health risk assessment was carried out using fixed exposure parameters, including hand-oral ingestion and dermal and respiratory exposure times, in combination with pollutant concentrations [45,46]. However, due to the randomness and incompleteness of environmental data, discrepancies may arise between the ecological risk assessment and actual results. In addition, human health risk evaluation conducted only through the model recommended by the United States Environmental Protection Agency (USEPA) can lead to a decrease in the reliability of the results of human health risk evaluation due to differences in parameters such as individual age, physical condition, gender, and metabolism [47,48].

As the largest inland still-water lake in Hunan Province and a vital part of Dongting Lake, Datong Lake serves multiple functions, including ecological nourishment, flood control and storage, and agricultural irrigation, among others. Its environmental status is crucial [49,50]. From 2010 to 2015, the water quality of Datong Lake deteriorated due to excessive baiting, fishing, and agricultural surface source pollution, as well as sediment. Sediment nitrogen and phosphorus loads exceeded the standard, and heavy metals such as Cd, Mn, and Cu had polluted the water, resulting in a degraded ecosystem [51]. Since 2017, water pollution and environmental and ecological remediation efforts have been undertaken in the lake area to establish a long-term protection mechanism. The water quality has continued to improve, and the nitrogen and phosphorus loads have been significantly reduced after 2023 [52]. However, the status of heavy metal pollution remains unknown.

It is hypothesized that (1) the heavy metal concentrations in the sediments of the study area exceed the background values of Dongting Lake (levels of natural content of various chemical elements or physical properties in sediments under natural conditions not significantly disturbed by human activity) [53], (2) the heavy metal contamination level of the sediments in the study area is high, and (3) the heavy metals in the sediments of the study area pose health threats to different populations. To verify the above hypotheses, this paper uses the sediments of Datong Lake as the research object, measures the content of eight heavy metals, including Cd, As, and Pb, and conducts a related evaluation of heavy metal pollution. To reduce uncertainty in the traditional risk assessment process and increase the reliability of assessment results [54], the probability distributions of pollutant concentrations and human exposure parameters should be considered and introduced into the risk assessment. Therefore, we introduced a Monte Carlo simulation method to assess the potential ecological hazards and health risks associated with heavy metals in sediments in the study area. This study employs a Monte Carlo simulation approach, grounded in the probability distribution modeling of uncertain parameters derived from measured data. Through multiple iterations of random sampling, this method addresses parameter uncertainty. We utilize the Geo-accumulation index and Enrichment factor method to characterize the heavy metal contamination in sediments within the study area. Subsequently, ecological risk and human health risk assessments are conducted to determine the environmental risk level and evaluate potential health threats posed by the current pollution status to different population groups. This work aims to provide a comprehensive and scientifically robust basis for ecological conservation and resident health protection in the Datong Lake area.

## 2. Materials and Methods

### 2.1. Overview of the Study Area

Datong Lake is located in Yiyang City, Hunan Province, in the hinterland of the Dongting Lake area, at 112°25′ E–112°35′ E, 29°7′30″ N–29°15″ N. The lake covers an area of approximately 75.30 square kilometers, with an average depth of around 2.5 m. The lake area falls within the monsoon-humid climate of the middle sub-tropical to the north sub-tropical region, with an average annual temperature of 16 °C. The yearly water temperature ranges between 10 and 15 °C. The average annual temperature is 16.6 °C, with an annual water temperature range of 10 to 32 °C.

### 2.2. Sample Collection and Measurement

**Figure 1:**
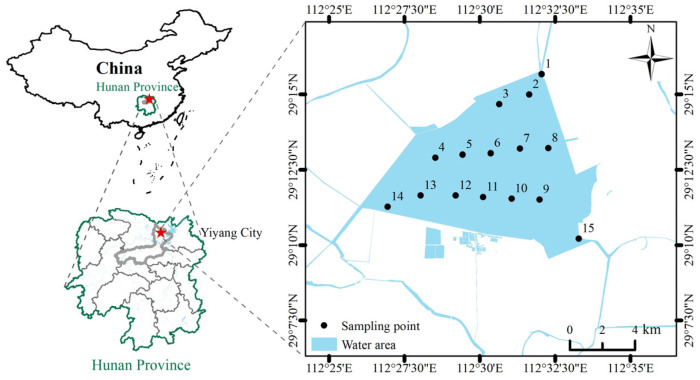
Map of the geographic location and sampling distribution of Datong Lake.

Previously, we conducted a pre-study of pollution sources in the Datong Lake watershed. Combining this with monitoring data from the Hunan Provincial Ecological and Environmental Department from 2019 to 2023, we found that there were fewer industrial enterprises around Datong Lake, and the management measures for agricultural surface pollution were effective. There was almost no point-source pollution, and the spatial distribution of pollutants was relatively uniform [55]. Meanwhile, to meet the needs of constructing the Monte Carlo model below, 15 sediment sampling points were established in this study (Figure 1). The sampling strictly followed the basic requirements for sampling heavy metals in lake sediments in the “Technical Specification for Surface Water and Wastewater Monitoring” [29] (HJ/T 91-2002). The sampling points were arranged at three outlets and inlets of Datong Lake to capture the input and output of exogenous pollutants. The center of the lake area was covered using a grid of uniformly distributed points to ensure that the main lake area could be adequately sampled.

Surface sediment samples (0–10 cm depth) were collected using a Peterson grab sampler (Zhejiang Yifeng Instrument Center, Zhejiang Province, Hangzhou, China) and stored in pre-cleaned self-sealing bags. Geographic coordinates and ambient environmental conditions at each sampling site were contemporaneously documented. Following transport to the laboratory, the samples underwent debris removal (including aquatic biota remnants and macrophyte roots) prior to freeze-drying. After air-drying, grinding, and sieving (100 mesh), the soil samples to be tested were accurately weighed to approximately 0.1000 g in a polytetrafluoroethylene (PTFE) ablation tank, and appropriate amounts of concentrated HNO_3_ and HF were added. The samples were then programmed to elevate the temperature for ablation using a microwave ablator. After digestion was completed, the solution was transferred to a polypropylene volumetric flask, and an appropriate amount of internal standard solution (Rh and Re elements were selected) was added. The volume was then adjusted to the scale with ultrapure water, and the flask was shaken well for storage. The iCAP-Q inductively coupled plasma mass spectrometer (iCAP-Q ICP-MS) produced by Thermo Fisher Scientific (Room 405, Block 4, 222 Meiyao Road, Shanghai Pilot Free Trade Zone, Shanghai, China) was used for the online test. The standard reference material (SRM 2704, lake bottom sediment) [56] provided by the National Institute of Standards and Technology (NIST) was used to draw the calibration curve. The instrument operating parameters were optimally set (RF power, nebulizing gas flow, sampling depth, and collision cell gas flow). In order to assess the precision and accuracy of the method, reagent blanks and parallel samples were inserted throughout the analytical process, and the method was validated using GBW07310 (standard substance for analyzing the composition of offshore marine sediments). The relative standard deviations (RSDs) were calculated by repeating the determination of the same samples (*n* = 10). The RSDs of each element were less than 5%, and the relative errors (REs) between the measured and certified values of each element in the GBW07310 standard substance ranged from −3.2% to +2.8%. In addition, the accuracy was assessed by spiking recovery experiments, in which mixed standard solutions of known concentration were added to the sediment samples, and the recoveries of each element were measured to be 92.5–105.3%. The sample solution was atomized to form an aerosol, which was then subjected to high-temperature ionization in a plasma. The target element ions were separated by a four-stage rod mass analyzer according to the mass-to-charge ratio (*m*/*z*) (As: 75, Cd: 111, Cr: 52, Cu: 63, Mn: 55, Ni: 60, Pb: 208, and Zn: 66), and the isotopic signal intensities of the ions were measured by a detector, which were calculated by combining with calibration curves—the content of each element in the soil samples [57].

### 2.3. Heavy Metal Pollution Evaluation Methods

#### 2.3.1. Geo-Accumulation Index Method

In the 1960s, the German scientist Muller proposed the Geo-accumulation index (*I_geo_*), which integrates the effects of human disturbance and natural factors on heavy metals in water sediments. The calculation formula is shown below [58]:

*I_geo_* = *log*_2_[*C*/(1.5*C*_0_)]

where *I_geo_* is the Geo-accumulation index of heavy metals; *C* is the actual content of heavy metals in sediment samples in mg/kg; *C*_0_ is the background value of heavy metals in the soil in mg/kg; in this paper, we chose the background value of the soil in Dongting Lake basin, Hunan Province, China, as the standard for calculation [59]; 1.5 is a constant set to correct for the disturbance of the background value of the environment by the effect of rock-forming; and the contamination strength of the Geo-accumulation index is shown in Table 1. The grading table is shown in Table 1.

#### 2.3.2. Enrichment Factor Method

The Enrichment factor (*EF*) method can reflect the enrichment of heavy metals in sediments by evaluating the Enrichment factor, which fully considers the influence of sediment particle composition and is calculated by the following formula [60]:

$$EF=\frac{{(C}_{i}/C_{r}{)}_{sample}}{(C_{i}/C_{r}{)}_{background}}$$

where ${(c}_{i}/c_{r}{)}_{sample}$ and $(c_{i}/c_{r}{)}_{background}$ are the measured values of element *i* and the background values of soil elements in the Dongting Lake basin (mg/kg), and $c_{r}$ is the content of the reference element (mg/kg). Since Al is one of the most extensive constituents of the earth’s crust, as well as being chemically stable, with low volatility, and less affected by human activities [60], Al was chosen in this study as the reference element. The grading table is shown in Table 2.

#### 2.3.3. Potential Ecological Risk Evaluation Methodology

The potential hazardous effects caused by a specific element correspond to its concentration and toxicological properties, and the potential ecological risk index method is used to calculate the toxic response of heavy metal elements in the environment and thus to assess the risk of the element, which is calculated as follows [61]:

$$C_{f}^{i}=c_{i}/c_{n}^{i}\;E_{f}^{i}=T_{r}^{i}/C_{f}^{i}\;RI=\sum_{i=1}^{n}E_{r}^{i}$$

where $C_{f}^{i}$ and $c_{i}$ are the pollution index and measured content of heavy metal element *i*, respectively; $c_{n}^{i}$ is the background value of the soil element in Hunan Province, China for element *i*, $E_{f}^{i}$ and $T_{r}^{i}$ are the potential ecological risk index and biological toxicity factor of element *i*, respectively; the toxicity factors of As, Cd, Cr, Cu, Pb, Zn, Mn and Ni in this study were taken as 10, 30, 2, 5, 5, 1, 1 and 5 [61]. *RI* is the index of the potential ecological risk of each heavy metal element combined (Table 3).

### 2.4. Health Risk Evaluation Methodology

The USEPA-recommended human health risk model was used to evaluate the non-carcinogenic and carcinogenic health risks for adults (male and female) and children active in the Datong Lake wetland. Considering that the possibility of respiratory uptake of heavy metals from wetland sediments is extremely low, only two modes of ingestion and dermal exposure were evaluated in the present study, which was calculated by the following formula [60]:

$${CDD}_{ing}=\frac{c^{k}\times IR_{ing}\times ED\times EF\times CF}{BW\times AT}\;{CDD}_{derm}=\frac{c^{k}\times SA\times AF\times ABS\times ED\times EF\times CF}{BW\times AT}$$

where ${CDD}_{ing}$ and ${CDD}_{derm}$ are the average daily exposure (mg/kg·d) by the ingestion and dermal contact routes, respectively, $c^{k}$ is the measured concentration of element *k* (mg/kg), $IR_{ing}$ is the ingestion rate, *EF* is the frequency of exposure, *ED* is the duration of exposure, *BW* is the body weight of the exposed individual, *AT* is the average time of exposure, *AF* is the skin adhesion factor, *SA* is the skin exposure surface area, *ABS* is the skin absorption factor, and *CF* is the unit conversion factor.

The non-carcinogenic risk of heavy metals in Datong Lake sediments to different populations was calculated as follows [61]:

$$HI=\sum HQ_{ij}=\sum \frac{{CDD}_{ing}}{RfD_{ij}}$$

where *HI* is the total non-carcinogenic risk, and $HQ_{ij}$ and $RfD_{ij}$ are the non-carcinogenic risk value and reference dose value of heavy metal *i* under exposure pathway *j*, respectively. If *HI* (or *HQ*) > 1, it means that the sediment heavy metal may pose a non-carcinogenic risk to human beings, and if *HI* (or *HQ*) ≤ 1, it means that the heavy metal in the sediment poses little non-carcinogenic risk to human beings.

The carcinogenic risk of heavy metals in Datong Lake sediments to different populations was calculated as follows [62]:

$$TCR=\sum CR_{ij}=\sum {CDD}_{ij}\times {SF}_{ij}$$

where *TCR* is the total carcinogenic risk, and $CR_{ij}$ and ${SF}_{ij}$ are the carcinogenic risk value and carcinogenic slope factor value of heavy metal *i* under exposure pathway *j*, respectively. The non-carcinogenic parameters and carcinogenicity slope factors of heavy metals are shown in Table 4. If *TCR* (or $CR_{ij}$) < 1 × 10^−6^, it means that the heavy metals in the sediments pose little or no carcinogenic risk to the human body; if 1 × 10^−6^ ≤ *TCR* (or $CR_{ij}$) < 1 × 10^−4^, it means that there is an acceptable carcinogenicity risk; and if *TCR* (or $CR_{ij}$) > 1 × 10^−4^, there is an unacceptably high carcinogenicity risk. The main parameters required for health risk assessment of different populations are shown in Table 5.

### 2.5. Monte Carlo Modeling

To overcome the over- or underestimation of traditional heavy metal pollution assessments caused by fixed parameters (toxicity response coefficients, background values, and exposure risks), this study applied a Monte Carlo simulation for pollution risk assessment and predictive analysis. The procedure included (1) constructing probability density distribution models; (2) defining distribution characteristics of each heavy metal; (3) performing model calculations with extensive random sampling; (4) statistical analysis of simulated data. The simulation used 10,000 random sampling iterations at a 95% confidence level (CI).

## 3. Results

### 3.1. Characterization of Sediment Heavy Metal Content

As shown in Table 6, the best-fit distributions of As, Pb, Cd, Cr, Cu, Mn, Ni, and Zn were obtained by the Anderson–Darling test (N): logistic distributions for Cd and Pb, minimum extreme distributions for Cr and Cu, maximum extreme distributions for As and Mn, triangular distributions for Ni, and binomial distributions for Zn.

From the results of heavy metal detection in Datong Lake sediments, it is clear that the range of concentrations of each heavy metal spans a small range. Compared with the background value of the Dongting Lake sediment-water system [63], the average concentrations of heavy metals at the sampling sites were 2–2.7 times higher than the background value of the Dongting Lake sediment-water system, except for heavy metal As.

### 3.2. Characterization of Heavy Metal Contamination of Sediments

#### 3.2.1. Geo-Accumulation Index

**Figure 2:**
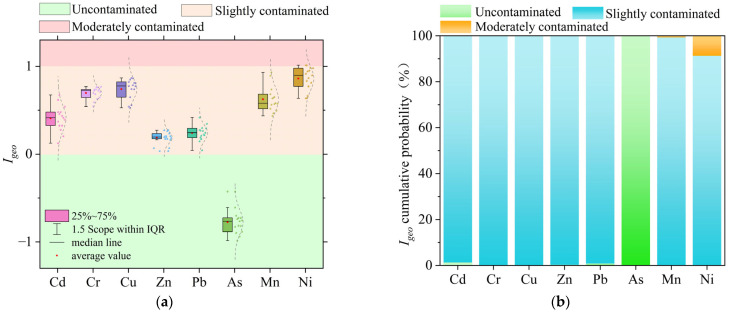
Evaluation results of the Geo-cumulative pollution index and probability: (**a**) results of the evaluation of the Geo-cumulative pollution index; (**b**) Geo-cumulative contamination probability from Monte Carlo modeling.

The average *I_geo_* value of each heavy metal element in the sediments of Datong Lake was calculated by the Geo-accumulation index as Ni (0.86) > Cu (0.74) > Cr (0.70) > Mn (0.63) > Cd (0.41) > Pb (0.24) > Zn (0.19) > As (−0.77). According to the *I_geo_* pollution level grading scale, all heavy metal elements are slightly pollutedexcept for As, which is not polluted. The predicted mean value of the sediment heavy metal *I_geo_* based on a Monte Carlo simulation is as follows: Ni (0.86) > Cu (0.75) > Cr (0.70) > Mn (0.62) > Cd (0.40) > Pb (0.24) > Zn (0.19) > As (−0.78), which, combined with Figure 2b, shows that the probability of As being uncontaminated is 99%. The probability that the remaining heavy metal elements are slightly polluting is above 90%, which is consistent with the evaluation results of the Geo-cumulative pollution index.

#### 3.2.2. Enrichment Factor

**Figure 3:**
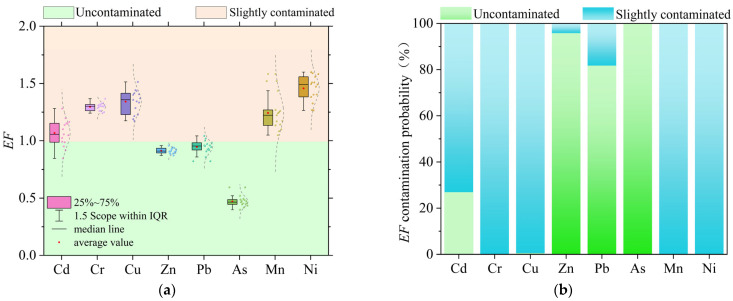
Evaluation results of Enrichment factors and probability: (**a**) results of Enrichment factor evaluation; (**b**) Monte Carlo-modeled probability of enriched contamination.

The enrichment calculations showed that the average *EF* values of each heavy metal element in the sediments of Datong Lake were Ni (1.46) > Cu (1.34) > Cr (1.30) > Mn (1.24) > Cd (1.07) > Pb (0.95) > Zn (0.91) > As (0.47). According to the *EF* contamination classification criteria, Pb, Zn, and As are classified as non- contaminated, while Ni, Cu, Cr, Mn, and Cd are classified as slightly contaminated. The predicted mean values of the sediment heavy metal *EF* based on the Monte Carlo simulation were Ni (1.46) > Cu (1.34) > Cr (1.30) > Mn (1.24) > Cd (1.06) > Pb (0.95) > Zn (0.91) > As (0.47). Combined with Figure 3b, this shows that the probability of Pb, Zn, and As being non- polluted is above 80%, and the likelihood of the rest of the heavy metal elements being slightly polluted s above 70%, which is generally consistent with the results of the Enrichment factor evaluation.

### 3.3. Evaluation of Potential Ecological Risks of Sediment Heavy Metals

**Figure 4:**
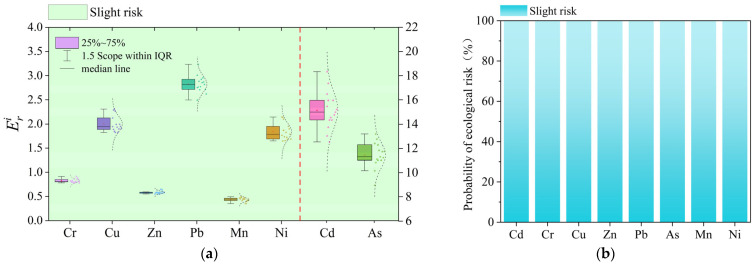
Combined potential risk and probability evaluation results: (**a**) consolidated potential risk evaluation results; (**b**) combined potential risk results from Monte Carlo modeling.

Calculation of the potential risk value shows that the average value of $E_{f}^{i}$ for each heavy metal element in the sediments of Datong Lake is as follows: Cd (15.15) > As (11.44) > Pb (2.83) > Cu (2.00) > Ni (1.84) > Cr (0.82) > Zn (0.59) > Mn (0.43), and all the heavy metals are at the level of slight pollution. The mean value of *RI* was 32.83, which is a slight ecological risk level. The predicted mean value of the sediment heavy metal $E_{f}^{i}$ based on the Monte Carlo simulation was Cd (15.16) > As (11.45) > Pb (2.83) > Cu (1.99) > Ni (1.84) > Cr (0.82) > Zn (0.58) > Mn (0.43), which, combined with Figure 4b, shows that all the heavy metal elements fall under slightly polluting, which is consistent with the above evaluation results.

### 3.4. Human Health Evaluation

#### Carcinogenic and Non-Carcinogenic Health Risk Evaluation

**Figure 5:**
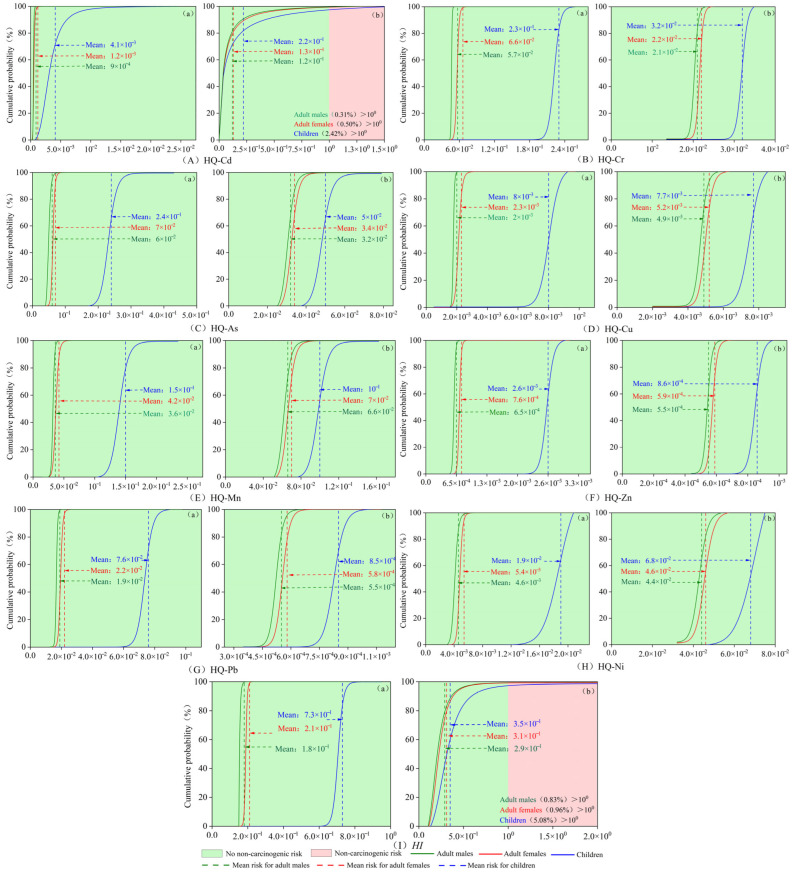
(**A**–**I**) Probabilistic non-carcinogenic health risk assessment for each heavy metal and all heavy metals. (**a**) Dermal exposure to heavy metals; (**b**) exposure to heavy metals by ingestion.

As shown in Figure 5A–I, there is no non-carcinogenic risk in different populations through the intake of elemental Cd. Through dermal exposure to elemental Cd, the probability that Cd poses a non-carcinogenic risk to adult males and females is less than 1%, and the likelihood that elemental Cd poses a non-carcinogenic risk to children is 2.42%. Except for Cd, none of the heavy metal elements exceeded the non-carcinogenic risk threshold (*HQ* = 1) by ingestion and dermal exposure modes. Therefore, Cd is the central element that exposes populations in the Datong Lake watershed to non-carcinogenic health risks.

The *HI* values for each heavy metal for adult males, females, and children, based on ingestion, were 0.18, 0.21, and 0.73, respectively. These *HI* values are less than 1, indicating that there is no non-carcinogenic risk associated with this type of exposure. Dermal exposure to heavy metals resulted in *HI* values of 0.29, 0.31, and 0.35 for adult males, females, and children, respectively. Notably, 0.83% of adult males, 0.96% of adult females, and 5.08% of children had *HI* values greater than one. This confirms a low but detectable non-carcinogenic risk associated with this exposure pathway. Notably, the Hazard Index (*HI*) across population groups followed a consistent hierarchy for both exposure routes: children > adult females > adult males. This elevated susceptibility in children is primarily attributable to their higher heavy metal absorption rates, lower metabolic detoxification capacity, increased relative exposure dose per unit body weight, and risk-prone behavioral patterns [64].

**Figure 6:**
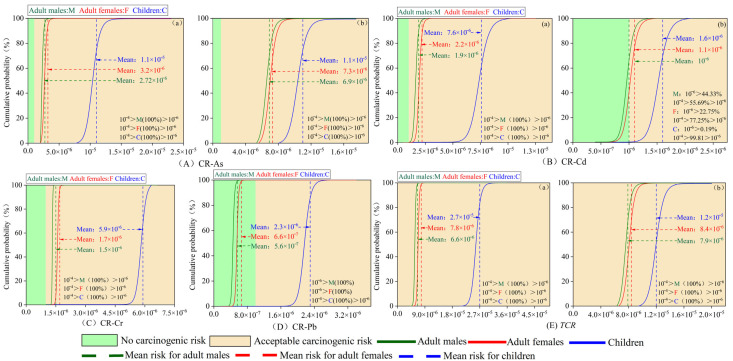
(**A**–**E**) Health risk assessment of carcinogenic probability for each and all heavy metals. (**a**) Dermal exposure to heavy metals; (**b**) exposure to heavy metals by ingestion.

The cumulative probability curves of the carcinogenic risk of each sediment heavy metal to different populations are shown in Figure 6. Overall, the carcinogenic risk results for the four heavy metals, As, Cd, Cr, and Pb, calculated from different exposure modes and populations, did not exceed the threshold values (*CR* < 10^−4^). The results of the carcinogenic risk of arsenic (As) to different groups under both exposure modes were in the range of 1 × 10^−6^ to 1 × 10^−4^, indicating that elemental As may pose a specific carcinogenic risk to various populations. Still, these risks were all within the acceptable range. Under the ingestion mode of heavy metals, the carcinogenic risk results of Cd for different groups were in the range of 1 × 10^−6^ to 1 × 10^−4^. Under dermal exposure to heavy metals, Cd did not pose a carcinogenic risk to 44.33% of adult males, 22.75% of adult females, and 0.19% of children, while 55.69% of adult males, 77.25% of adult females, and 99.81% of children, respectively, had an acceptable carcinogenic risk. Due to the limitations of data and modeling, this paper calculated the effect of heavy metals on human carcinogenic risk only under ingestion conditions for Cr and Pb elements. From the results, it can be seen that the carcinogenic risk results for Cr in different groups were in the range of 1 × 10^−6^ to 1 × 10^−4^. Pb did not pose a carcinogenic risk to adult males and adult females, but it may pose a carcinogenic risk to children, which is within the acceptable range. The magnitude of the mean *CR* of each heavy metal for different groups under the ingestion mode of heavy metals was As > Cd > Cr > Pb.

## 4. Discussion

### 4.1. Discussion of the Causes of Heavy Metal Contamination of Sediments

#### 4.1.1. Impact of Human Activities

**Figure 7:**
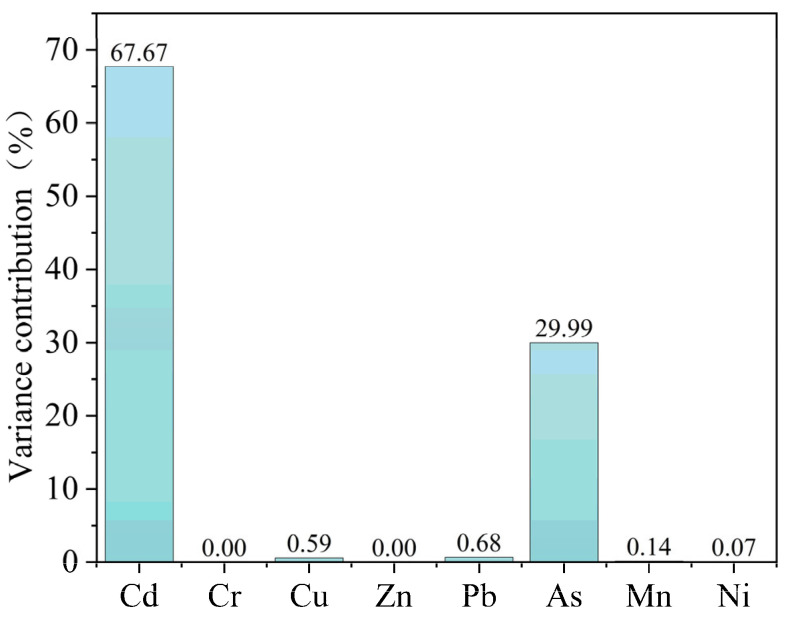
Monte Carlo-simulated contribution of heavy metals to the combined potential ecological risk.

Sediments in Datong Lake were generally slightly contaminated. We further understood the impact of each heavy metal on ecological risk through sensitivity analysis. As shown in Figure 7, cadmium and arsenic are the main factors contributing to the risk of heavy metals in the sediments of Datong Lake. The main reason for this is that the toxicity coefficient of heavy metal elements mainly determines the potential ecological risk. Among other heavy metals, Cd and As have the highest toxicity coefficients and therefore contribute the most to the ecological risk [65]. Cadmium concentrations in Dongting Lake were significantly higher than background values, which may be closely related to regional human activities [50]. Comparative studies of other lakes in the middle and lower reaches of the Yangtze River (e.g., Tai Lake and Chaohu Lake) found [66,67] that Datong Lake generally has a low level of heavy metal pollution. However, cadmium (Cd) contributed prominently to the ecological risk, which was related to historical residual pollution from non-ferrous metal mining and smelting activities in the Dongting Lake basin [68]. In addition, the average concentration of the heavy metal arsenic (As) was 0.9 times the background value. However, it still exceeded the standard, which may be related to regional pesticide use or substrate release [69]. From the coefficient of variation, it can be seen that the coefficient of variation of manganese was the largest, which was 10.30%, indicating that the variability of each heavy metal was small, and the spatial distribution of the content of each heavy metal was relatively homogeneous, and the possibility of the existence of point-source pollution was small.

#### 4.1.2. Regulation by Natural Factors

The organic matter content of sediments in Datong Lake is higher than the average level in the middle and lower reaches of the Yangtze River [70]. Its functional groups can adsorb up to 30–50 mg/g of Cd and Pb, which significantly reduces their bioavailability [71]. However, acidic conditions (pH 5.8–6.2) led to increased release of Mn, which explains why Mn (mean value 1046.13 mg/kg) far exceeded the background value (450 mg/kg). Under localized anaerobic conditions, Mn (IV) was reduced to soluble Mn (II), which was subsequently reoxidized and redeposited after migrating with pore water, resulting in fluctuating concentrations [72].

### 4.2. Major Factors Affecting Human Health in the Datong Lake Area

#### 4.2.1. Main Factors Influencing the Health Risks of Different Population Groups in the Datong Lake District

The mean *HI* and *TCR* values of heavy metals in the sediments of Datong Lake indicated that the non-carcinogenic and carcinogenic risks of different populations were within acceptable limits. Children had the highest probability of health risk, mainly due to their lower resistance to toxic substances [64]. Combined with the results of previous ecological risk assessments, although the carcinogenic risk of heavy metals in the sediments of Lake Datong to different populations is low, continuous monitoring and attention to changes in the elements of cadmium and arsenic are still needed [8].

It should be noted that this study used the USEPA-recommended health risk evaluation model to assess the health risk of sediments in Datong Lake, and the parameters cited are all based on the general parameters recommended by the USEPA, which may differ from the specific conditions of the Datong Lake area and thus have some limitations [73]. Additionally, the evaluation of health risks associated with ingestion and dermal exposure to heavy metals is closely tied to factors such as gender, age, and occupation type [74]. Local fishermen and farmers are predominant in the Datong Lake area [50], and this group of people requires more sophisticated methods to obtain exposure doses of heavy metals for different groups of people. Therefore, the health risk assessment methods of heavy metals in the sediments of Datong Lake still need to be further explored and improved in follow-up research work.

#### 4.2.2. Sensitivity Discussion of Human Health Risk Parameters

**Figure 8:**
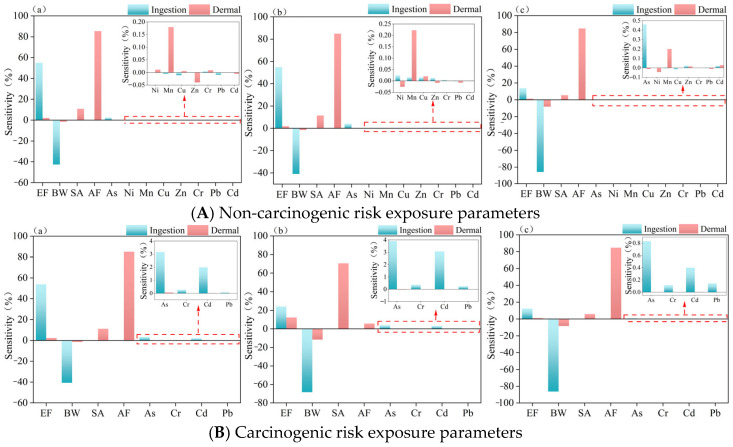
(**A**,**B**) Sensitivity map for probabilistic assessment of non-carcinogenic versus carcinogenic health risks. (**a**) Adult males; (**b**) adult females; (**c**) children.

As illustrated in Figure 8, sensitivity analysis of the health risk evaluation model parameters was conducted based on prior risk assessment results. The non-carcinogenic risk assessment revealed that the eight target heavy metals contributed less than 1% to total risk, underscoring the predominant influence of physiological parameters. For the ingestion pathway, exposure frequency (EF) exhibited a significant positive correlation with health risk (sensitivity coefficient: 54% for adults), whereas body weight (BW) demonstrated an inverse relationship. Children showed substantially greater sensitivity to BW changes (sensitivity coefficient: −86%) compared to adults (males: −43%; females: −41%). Under dermal exposure, the skin adherence factor (AF) consistently exhibited high sensitivity coefficients (up to 85%), confirming a strong association between pollutant adherence properties and exposure behavior [75]. Carcinogenic risk analyses further identified As and Cd as primary risk sources, with exposure patterns highly consistent with non-carcinogenic risks. Sensitivity analysis indicated that increased body weight correlated with reduced susceptibility to toxic substances, warranting consideration of access controls for children in lake wetland areas—a finding consistent with Chen et al. [76]. Dermal exposure pathways and contaminant adherence characteristics represent critical focus areas. These results further indicate the need for refined AF parameterization in future studies to enhance health risk assessment accuracy.

## 5. Conclusions

In this paper, the sediments of Datong Lake in Hunan Province were selected as the research object. By collecting soil samples from the surface layer of the sediments, determining the contents of soil heavy metals Cd, As, Pb, Cr, Cu, Mn, Pb, Ni, and Zn, and applying various evaluation methods to study the heavy metal contents and pollution characteristics of the sediments, we further assessed potential ecological hazards and probability of risk to human health due to heavy metal contamination. We came up with the results and conclusions as follows:

(1) The average values of Cd, Pb, Cr, Cu, Mn, Pb, Ni, and Zn contents in the sediments of Datong Lake were higher than the background values of soil elements in the sediments of Dongting Lake. The average value of As content in heavy metals was lower than the background value of soil, and the possibility of point-source contamination in the study area is slight.
(2) According to the evaluation results of the Geo-cumulative index method and Enrichment factor method, the average values of *I_geo_* and *EF* of heavy metal elements in the sediments of Daitong Lake were Ni > Cu > Cr > Mn > Cd > Pb > Zn > As, and the grading standards of the *I_geo_* pollution level showed that all heavy metal elements were slightly polluted except As, which was not polluting. From the *EF* pollution level grading standard, Pb, Zn, and As are non- polluted, and Ni, Cu, Cr, Mn and Cd are slightly polluted. The combination of the two evaluation methods can verify each other and make up for the limitations of a single method.
(3) The analysis of potential risk shows that the average value of $E_{f}^{i}$ for each heavy metal element in the sediments of Datong Lake was as follows: Cd > As > Pb > Cu > Ni > Cr > Zn > Mn, and all heavy metal elements are slightly polluted. Among them, Cd and As are the main contributing elements to the risk of heavy metals in the sediments of Datong Lake.
(4) Both non-carcinogenic and carcinogenic risks for all populations in the study area remain low for heavy metal exposure via ingestion and dermal pathways. In the ecological and health risk assessments, As and Cd exhibited significantly higher sensitivity than the other heavy metals. Consequently, continuous monitoring and source-tracking of Cd and As are essential to safeguard long-term ecological integrity and public health in the region.

## Figures and Tables

**Table 1:** *I_geo_* Contamination level classification criteria.

Level	Pollution Index	Pollution Category
0	≤0	Uncontaminated
1	(0,1]	Slightly
2	(1,2]	Moderately
3	(2,3]	Moderately/Heavily
4	(3,4]	Heavily
5	>4	Extremely

**Table 2:** *EF* contamination level classification criteria [59].

Level	Pollution Index	Pollution Category
0	≤1	Uncontaminated
1	(1,2]	Slightly
2	(2,5]	Moderately
3	(5,20]	Moderately/Heavily
4	(20,40]	Heavily
5	>40	Extremely

**Table 3:** Criteria for classifying the degree of RI contamination.

Level	Pollution Index	Risk Class
0	≤150	Slight risk
1	(150,300]	Moderate risk
2	(300,600]	Higher risk
3	(600,1200]	High risk
5	>1200	Extremely high risk

**Table 4:** Heavy metal reference dose (RfD) and slope factor (SF) for health risk assessment.

Elements	RfD		SF	
	Ingestion	Dermal	Ingestion	Dermal
As	3.00 × 10^−4^	1.23 × 10^−4^	1.50 × 10^0^	1.50 × 10^0^
Cd	1.00 × 10^−3^	1.00 × 10^−5^	1.80 × 10^0^	3.80 × 10^−1^
Cr	3.00 × 10^−3^	6.00 × 10^−5^	5.00 × 10^−1^	-
Cu	4.00 × 10^−2^	1.20 × 10^−2^	-	-
Hg	3.00 × 10^−4^	2.10 × 10^−5^	-	-
Ni	2.00 × 10^−2^	5.40 × 10^−3^	-	-
Pb	3.50 × 10^−3^	5.25 × 10^−3^	8.50 × 10^−3^	-
Zn	3.50 × 10^−1^	6.00 × 10^−2^	-	-
Mn	4.60 × 10^−2^	1.84 × 10^−3^	-	-

**Table 5:** Distribution of probability parameters for health risk assessment of heavy metals in sediments.

Exposure Parameters	Unit	Probability Distribution	Adult Males	Adult Females	Children
IRing	mg/d	point	114	114	200
ED	a	point	70	70	18
EF	d/a	triangular	345 (180–365)	345 (180–365)	345 (180–365)
BW	kg	logarithmic	67.55 ± 8.72	57.59 ± 8.03	-
BW	kg	triangular	-	-	29.30 (5.20–56.80)
ABS	-	point	0.03 (As), 0.14 (Cd), 0.001 (Cr), 0.1 (Cu), 0.35 (Ni), 0.006 (Pb), 0.02 (Zn), 0.01 (Mn)		
SA	m^2^	triangular	0.169 (0.085–0.422)	0.153 (0.076–0.382)	0.086 (0.043–0.216)
AF	mg/cm^2^·d	logarithmic	0.49 ± 0.54	0.49 ± 0.54	0.65 ± 1.2
CF	-	point	10 (^−6^)	10 (^−6^)	10 (^−6^)
AT (non-carcinogenic)	d	point	365 × ED	365 × ED	365 × ED
AT (carcinogenic)	d	point	365 × 70	365 × 70	365 × 70

Triangular distribution: most probable value (minimum, maximum); logarithmic distribution: mean ± standard deviation.

**Table 6:** Characteristics of heavy metal content in wetland sediments of Datong Lake.

	Cd	Cr	Cu	Zn	Pb	As	Mn	Ni
Min/(mg/kg)	0.54	96.20	43.70	128.00	36.00	9.78	914.00	49.40
Max/(mg/kg)	0.79	112.42	55.30	151.00	46.70	14.40	1287.00	64.20
Median/(mg/kg)	0.66	109.00	51.90	143.00	41.38	11.40	1010.00	59.27
Mean/(mg/kg)	0.66	107.07	50.81	142.67	41.36	11.37	1046.13	57.99
SD	0.07	4.85	3.98	6.44	2.51	1.13	107.80	5.15
CV/%	10.13	4.53	7.84	4.52	6.08	9.94	10.30	8.89
DLSHBV/(mg/kg)	0.33	44.00	20.20	83.30	23.30	12.90	450.00	21.20
TF	30	2	5	1	5	10	1	5
Distribution type	logistic	minimum extreme	minimum extreme	binomial	logistic	maximum extreme	maximum extreme	triangular

## Data Availability

Data will be made available on request.

## Abbreviations

The following abbreviations are used in this manuscript:

USEPA	United States Environmental Protcction Agency
SD	Standard Deviation
CV	Coefficient of Variation
DLSHBV	Dongting Lake sediment hydrological background values
TF	Toxicity factor
